# Psychometric properties of the questionnaire on threat perception of chronic illnesses in pediatric patients*

**DOI:** 10.1590/1518-8345.3144.3242

**Published:** 2020-02-03

**Authors:** Selene Valero-Moreno, Laura Lacomba-Trejo, Sara Casaña-Granell, Vicente Javier Prado-Gascó, Inmaculada Montoya-Castilla, Marian Pérez-Marín

**Affiliations:** 1 University of Valencia, Faculty of Psychology, Valencia, Comunidad Valenciana, Spain.

**Keywords:** Psycometrics, Endocrinology, Pediatrics, Pulmonary Medicine, Adaptation, Psychological, Psicometria, Endocrinologia, Pediatria, Pneumologia, Adaptação, Psicológica, Psicometría, Endocrinología, Pediatría, Neumología, Adaptación, Psicológica

## Abstract

**Objective:**

the objective of the study was to assess the psychometric properties of the Brief Illness Perception Questionnaire in a sample of adolescents with chronic endocrine or pneumological conditions and to analyze the dimensionality and reduce the scale elaborating scales by sex and medical diagnosis.

**Method:**

we evaluated 510 patients aged 9-16 years using the Brief Illness Perception Questionnaire and the Hospital Anxiety and Depression Scale. We carried out tests of reliability, construct and criterion validity and a comparison of means based on the diagnosis and socio-demographic variables. The reliability and validity analyses showed adequate psychometric properties for this scale, with better results obtained for a single dimension after eliminating 3 items.

**Results:**

adolescents with type 1 diabetes and girls were found to have an increased threat perception of their illness. Anxiety/depression was positively associated with the perception of illness.

**Conclusion:**

this questionnaire is a useful and practical tool for evaluating adjustment to illness in pediatric patients.

## Abstract (pt)

**Objetivo:**

o objetivo do estudo foi avaliar as propriedades psicométricas do Questionário de Percepção de Doenças Versão Breve em uma amostra de adolescentes com condições endócrinas ou pneumológicas crônicas. Analisar a dimensionalidade e reduzir a escala elaborando baremas por sexo e diagnóstico médico.

**Método:**

avaliamos 510 pacientes com idades entre 9 e 16 anos usando o Questionário de Percepção de Doenças Versão Breve e a Escala Hospitalar de Ansiedade e Depressão. Foram realizados testes de confiabilidade, validade do construto, validade de critério e comparação de médias segundo o diagnóstico e as variáveis sociodemográficas. As análises de confiabilidade e validade mostraram propriedades psicométricas adequadas para essa escala, obtendo melhores resultados para uma única dimensão após a exclusão de 3 itens.

**Resultados:**

observou-se que adolescentes com diabetes tipo 1 e meninas demonstraram maior percepção da ameaça da doença. A ansiedade/depressão associou-se positivamente à percepção da doença.

**Conclusão:**

este questionário é uma ferramenta útil e prática para avaliar o ajuste à doença em pacientes pediátricos.

## Abstract (es)

**Objetivo:**

el objetivo del estudio ha sido valorar las propiedades psicométricas del Cuestionario Breve de Percepción de la Enfermedad en una muestra de adolescentes con condiciones crónicas endocrinas o neumológicas. Analizar la dimensionalidad y reducir la escala elaborando baremos por sexo y diagnóstico médico.

**Método:**

evaluamos 510 pacientes de entre 9-16 años mediante el Cuestionario Breve de Percepción de la Enfermedad y la Escala Hospitalaria de Ansiedad y Depresión. Realizamos pruebas de fiabilidad, validez de constructo y criterial y comparación de medias en función del diagnóstico y variables sociodemográficas. Los análisis de fiabilidad y validez mostraron adecuadas propiedades psicométricas para esta escala, obteniendo mejores resultados para una única dimensión después de eliminar 3 ítems.

**Resultados:**

se observó que los adolescentes con diabetes tipo 1 y las niñas mostraban mayor percepción de amenaza de la enfermedad. La ansiedad/depresión se asociaron positivamente con la percepción de enfermedad.

**Conclusión:**

este cuestionario resulta una herramienta útil y práctica, con la que evaluar el ajuste a la enfermedad en pacientes pediátricos.

## Introduction

The evaluation of the perception of illness has aroused great interest^(^ 1 ^)^ as it may affect patients’ quality of life, well-being and adherence to treatment, among other factors^(^ 1 ^-^ 6 ^)^. Those who perceive their illness as more threatening, appear to present more anxiety, depression and a poorer quality of life^(^ 1 ^-^ 3 ^)^.

The processing of emotional and cognitive responses to illness helps patients to regulate themselves emotionally in order to better adapt to their condition, which can in turn change their cognitive or emotional representation. The term “perception of illness” therefore refers to a patient’s mental representations of their illness, in relation to its identity (name and symptoms), causes, consequences, course and control^(^ 7 ^-^ 8 ^)^.

All this seems particularly relevant in the case of chronic diseases (CD), since these are health problems that persist over time and requires continuous management and lifestyle changes over the years, and may affect the possible future evolution of the disease^(^ 9 ^)^.

However, not all CD appear to have the same effects. These effects seem to be more serious in the case of endocrine diseases, such as Diabetes Mellitus Type 1(DM1), than in respiratory diseases, such as asthma^(^ 10 ^)^.

Despite its importance, few studies have addressed this concept in adolescence, a particularly complicated period with lower rates of adherence to treatment, a greater presence of risk behaviors and more psychopathological problems^(^ 5 ^,^ 11 ^)^.

Although there are different models and instruments for the study of adaptive response to disease^(^ 12 ^-^ 13 ^)^, the prevailing model in the literature for some years has been the Common Sense Self- Regulation Model^(^ 14 ^)^.

Based on this model, the Illness Perception Questionnaire (IPQ)^(^ 10 ^)^ was designed, and is probably the most widely used instrument at international level for assessing this construct^(^ 1 ^,^ 15 ^)^. It initially had 38 items, and an extended revised version (IPQ-R) was subsequently designed, consisting of 70 items, including cognitive and emotional responses^(^ 16 ^)^. In recent years, in order to simplify and facilitate its application, the short IPQ (IPQ-R) was developed. This is composed of 9 items^(^ 10 ^)^ providing a quick and simple evaluation of this construct^(^ 17 ^)^.

The IPQ-R has been used in 36 countries and translated into 26 languages, and has been used in numerous diseases^(^ 2 ^-^ 3 ^,^ 18 ^-^ 19 ^)^ among patients aged 8 to 80 years old. The linguistic adaptation of IPQ-R to Spanish was carried out with adults with different CD, including endocrines and respiratory^(^ 15 ^)^. However, despite its widespread use, few studies analyze its psychometric properties^(^ 2 ^,^ 20 ^-^ 22 ^)^, and these are even more scarce in the Spanish context^(^ 15 ^)^. Likewise, there do not appear to be any studies analyzing the psychometric properties of IPQ-R pediatric patients with chronic conditions.

One aspect in relation to this instrument that continues to be a source of debate refers to its dimensionality. While some studies suggest using each item as a subscale^(^ 17 ^)^, others postulate the existence of two (cognition and emotion)^(^ 18 ^)^ or three dimensions (adding understanding of the disease to the two previous dimensions)^(^ 20 ^)^. Similarly, there seems to be no consensus on the effect that sex, age, or type of disease have on threat perception^(^ 10 ^,^ 23 ^-^ 24 ^)^. Another limitation of the instrument refers to the non-existence of interpretative scales that facilitate its interpretation depending on the type of disease or chronic condition.

The main objective of the study will therefore be to analyze the psychometric properties of BIP-Q in a sample of chronically ill adolescents, while studying the dimensionality of the scale and the effect of age, sex and medical diagnosis, and to offer interpretative scales.

This will all enable the development of intervention and prevention programmes that improve the quality of life of these patients.

## Method

The participants (n=510) were adolescents with chronic diseases between 9 and 16 years of age (M=12.03, SD=2.05). After obtaining the informed consent of the tutors, the data were collected between June 2016 and January 2018, the Endocrinology or Pediatric Pneumology Units of hospitals in the Valencian Community. This research has been endorsed by the Ethics Committees of the University of Valencia and the various participating hospitals.

The information was collected through an interview using an instrument composed of an ad hoc record with sociodemographic variables and two standardized instruments (BIP-Q and HADS) by the same professional in all cases. For the ad hoc record, the variables analyzed were age, sex of patient and type of pediatric chronic disease, and the instruments used to analyze the variables were the the Brief Illness Perception Questionnaire (IPQ-R) and the Hospital Anxiety and Depression Scale (HADS).

The Spanish version of the Brief Illness Perception Questionnaire (IPQ:R)^(^ 10 ^)^^(^ 15 ^)^ consists of 9 items. The first 8 items (consequences, duration, personal control, treatment control, identity, concern, emotional response and understanding of the disease) are answered on a Likert scale from 0 to 10, depending on the degree of agreement. The last item, causes, is in open response format, and assessed by citing the 3 most important responses believed to have caused the disease. The overall score is obtained by inverting items 3, 4 and 7 and adding them to the score for items 1, 2, 5, 6 and 8. The higher the total score, the greater the perception of the illness as a threat. The BIP-Q has been demonstrated to have adequate psychometric properties in previous studies not performed in adolescent populations. The questionnaire showed adequate internal consistency indices ranging from α=.67-.89, depending on the study and the type of sample^(^ 2 ^,^ 10 ^,^ 15 ^,^ 20 ^-^ 22 ^)^.

The version of the Hospital Anxiety and Depression Scale (HADS)^(^ 25 ^)^ adapted to Spanish^(^ 26 ^)^ is a Likert scale composed of 14 items grouped into two dimensions: anxiety (HADS-A) and depression (HADS-D). Both subscales are composed of 7 items interspersed throughout the questionnaire (Anxiety is the odd-numbered items and Depression is the even-numbered items). The response format ranges from 0 to 3, with 0 being the minimum score or no symptomatology, and 3 being the maximum score or presence of symptomatology. The higher the score, the higher the level of anxiety-depressive symptomatology. Previous studies have found adequate psychometric properties in relation to its reliability or internal consistency^(^ 26 ^)^: α=.68-.93 (Mα=.83) for the anxiety scale and for the depression scale between .67 and .90 (Mα=.82)^(^ 27 ^-^ 28 ^)^ generally obtaining higher scores on the anxiety scale. In this sample, α= .63 for anxiety and α=.56 for depression was obtained.

For data analysis: first, the reliability of the scale (Cronbach alpha, composite reliability (CF) and mean extracted variance index (MEVI)) was analyzed, and its validity (exploratory factorial analysis (EFA), confirmatory factorial analysis (CFA) and predictive validity of the instrument) was then analyzed. The EFA was performed using the recommended process^(^ 29 ^)^ using the unweighted least squares (ULS) method, parallel analysis and direct Oblimin rotation. The CFA were carried out by means of robust estimation of maximum likelihood (ML), with the objective of correcting the absence of multivariate normality. The suitability of the CFA was tested using the chi-square significance and robust Satorra-Bentler adjustments (S-B χ²)^(^ 30 ^-^ 31 ^)^, the ratio between χ² and its degrees of freedom (χ²/df), as well as the S-B X² and its degrees of freedom (proper setting≤5)^(^ 32 ^)^, the comparative setting index (CFI), the incremental setting (IFI) (proper setting≥.90)^(^ 33 ^)^ and the mean square error approach (RMSEA) (proper setting≤.08)^(^ 34 ^)^.

The relationship between BIP-Q and HADS was then analyzed using multiple hierarchical linear regressions, followed by differences in means according to the diagnosis (single factor ANOVA), sex and the two age groups of preadolescents (9-12 years) and adolescents (12-16 years) (t-test). They were recoded into two groups by age. Finally, scales were calculated based on centile scores according to diagnosis and sex. Statistical analyses were performed using SPSSv24, FACTOR^(^ 35 ^)^ software and EQSv6.3.

## Results

The participants (n=510) were minors with chronic diseases between 9 and 16 years old (M=12.03, SD=2.05), 42.4% (n=216) were preadolescents (9-12 years old) and 55.6% (n=294) were adolescents (12-16 years old). 54.5% of them were children (n=278). The sample was distributed as follows: 51.1% (n=262) presented chronic respiratory problems (PRC), 22.4% (n=113) had DM1 and 26.5% (n=135) had short stature (SS).

By analyzing the internal consistency of the BIP-Q scale, we found the initial reliability of the original structure for the unifactorial and bifactorial solution showed inadequate indices (α≤.70). In the final version of the questionnaire after eliminating three items, the data showed an improvement in the reliability for the unifactorial solution, complying with the acceptable indices (α=.76) but this improvement did not occur for the bifactorial solution (Table 1). Similarly, when calculating composite reliability (CF), only the indices in the unifactor solution were acceptable. Finally, when analyzing the mean variance extracted (IVE), the scores were adequate in both models as the scores were over .40. For all these reasons, the unifactorial solution was considered to present the best internal consistency indices.

**Table 1:** Analysis of items, reliability and IVE* of the questionnaire for each solution. Valencia, Spain, 2016-2018

Solution or model		M^†^	SD^‡^	r_jx_^§^	α-x^||^	A^¶^	K**
Unifactorial	Disease threat α^††^ = 0,56; α^††^ (without ítems 3, 4 and 7) = 0,76; ;CR^‡‡^=0,82; CI^§§^=(0,72-0,79); AVE*=0,57						
	BIP-Q1	3,16	2,52	0,52	0,45	0,50	-0,45
	BIP-Q2	5,71	3,08	0,19	0,53	-0,05	-1,23
	BIP-Q3	3,55	3,11	0,15	0,57	0,78	-0,46
	BIP-Q4	1,86	2,24	0,09	0,57	1,37	1,48
	BIP-Q5	3,28	2,73	0,34	0,50	0,37	-0,86
	BIP-Q6	3,65	3,01	0,50	0,44	0,45	-0,87
	BIP-Q7	3,70	3,06	0,05	0,63	0,62	-0,58
	BIP-Q8	2,59	3	0,45	0,46	0,87	-0,46
Bifactorial	Cognitive: α^††^ = 0,38; α^††^ (without ítems 3 and 4) = 0,62; ;CR^‡‡^=0,69; CI^§§^=(0,56-0,67); AVE*=0,59)						
	BIP-Q1	3,16	2,52	0,39	0,18	0,50	-0,45
	BIP-Q2	5,71	3,08	0,20	0,33	-0,05	-1,23
	BIP-Q3	3,55	3,11	0,06	0,45	0,78	-0,46
	BIP-Q4	1,86	2,24	0,11	0,39	1,37	1,48
	BIP-Q5	3,28	2,73	0,25	0,29	0,37	-0,86
	Afective: α^††^ = 0,68; α= 0,68 ;CR^‡‡^=0,84; CI^§§^=(0,62-0,73); AVE*=0,84)						
	BIP-Q6	3,65	3,01	0,51	-	0,45	-0,87
	BIP-Q8	2,59	3	0,51	-	0,87	-0,46

* AVE = Index of extracted variance (suitable ≥,40);

† M = mean;

‡ SD = Standard deviation;

§ r_jx_ = Item-total correlation;

|| α-x = Cronbach's alpha without the item;

¶ A = Asymmetry;

** K = Kurtosis;

†† α= Cronbach's alpha (suitable ≥,70);

‡‡ CR = Composite reliability (suitable ≥,70)

Prior to EFA and CFA, we determined the adequacy of the data using the Kaiser-Meyer-Olkin analysis (KMO) and Bartlett’s sphericity test. KMO analysis (KMO=.75) and Bartlett’s sphericity test (χ^2^=393.6, gl=28; p≤.001) obtained adequate values, so EFA and CFA were performed. Then, as suggested by the literature^(^ 29 ^-^ 30 ^)^, the sample was segmented into two groups, controlling for age, sex and diagnosis: group A (n=255) was used for EFA, group B (n=255) for CFA.

The EFA was calculated using FACTOR with the 8 items of the original version. Parallel analysis suggested two structures, with one formed by a single dimension and the other formed by two dimensions. After applying the EFA fixed to two dimensions, we decided to eliminate item 7 (coherence), since its factorial load was less than .40 (the specific value was 0.35). After this elimination, the factorial solution presented good adjustment indices (RMSEA=.05; CFI=.99), the variance was explained by the first factor of 23.66% and by the second factor of 11.46%. For the unifactorial dimension, the data suggested eliminating items 3, 4 and 7 because their factorial loads were below .40 (Item 3=.06; Item 4=0.02 and Item 7=0.24) (RMSEA=.12, IFC=.85), and the total variance was explained as 21.33%.

Various CFAs were then carried out. The goodness-of-fit indicators for the two solutions (unifactorial and bifactorial) were inappropriate, so it was necessary to re-specify the model by eliminating items (items 3, 4 and 7) (Table 2). After these re-specifications, both models presented a suitable fit although the fit indices were slightly better for the unifactorial solution. Finally, a reduced version of the 5-item questionnaire was obtained, with a final score of 50 points and the highest score related to the highest perception of illness threat (Figure 1).

**Table 2:** EFA* adjustment indicators for BIP-Q^†^ bifactorial and unifactorial solutions. Valencia, ESP, Spain, 2016-2018

Model	χ2^‡^	S-B-χ2^§^	*gl||*	*p¶*	S-B χ²/gl**	CFI^††^	IFI^‡‡^	RMSEA^§§^
8 items (1 factor)	120.34	83.46	20	≤0.001	4.17	0.77	0.78	0.11(0.09-0.14)
8 items (2 factors)	218.78	136.06	12	≤0.001	11.33	0.53	0.55	0.20 (0.17-0.23)
5 items (1 factor without ítems 7,4,3)	12.94	8.12	5	0.15	1.62	0.99	0.99	0.05 (0.01-0.11)
5 items (2 factors without ítems 7,4,3)	12.85	8.58	4	0.07	2.15	0.98	0.98	0.07 (0.01-0.13)

* EFA = Exploratory factor analysis;

† BIP-Q = Disease threat perception questionnaire;

‡ χ2 = chi-square;

§ S-B-χ^2^ = Robust correction of Satorra-Bentler chi-square;

| gl = Degrees of freedom;

¶ p = Level of significance;

** S-Bχ^2^/gl = Ratio between S-B χ^2^ and gl, Good fit≤5);

†† CFI = Comparative adjustment index (appropriate ≥,90);

‡‡ IFI = Bollet adjustment index (good fit≥0,90);

§§ RMSEA = Mean quadratic approximation error (good fit: ≤0.08)

**Figure 1:** Final version of the BIP-Q questionnaire* of this version. Valencia, ESP, Spain, 2016-2018

BIP-Q*
For the following questions, please circle the number that best represents your opinion.
**How much does your illness affect your life?**
(Doesn't affect you at all) 0 1 2 3 4 5 6 7 8 9 10 (Seriously affects my life)
**How long do you think your illness will last?**
(Very little time) 0 1 2 3 4 5 6 7 8 9 10 (Forever)
**To what extent do you feel symptoms due to your illness?**
(Absolutely nothing to worry about) 0 1 2 3 4 5 6 7 8 9 10 (Extremely)
**How worried are you about his illness?**
(Absolutamente nada preocupado) 0 1 2 3 4 5 6 7 8 9 10 (Extremamente preocupado)
**How emotional does your illness affect you? (That is, does it make you feel angry, scared, angry, or depressed?**
(Not emotionally affected at all) 0 1 2 3 4 5 6 7 8 9 10 (Extremely emotionally affected)

* BIP-Q = Disease Threat Perception Questionnaire

As suggested by the literature ^(^ 10 ^,^ 23 ^-^ 24 ^),^ we then analyzed the effect of age and the perception of threat of the illness (BIP-Q) on anxiety and depression, using two two-step hierarchical linear regressions using age (step 1) and the perception of threat of the illness (step 2) as predictor variables, and anxiety and depression measured by HADS as criterion variables. In the first step, age explained 3% (ΔR^2^=03, p≤.01) of anxiety and 2% (ΔR^2^=.02, p≤.05) of depression, inclusion of the perception of threat of the illness increased 3% (ΔR^2^=.03, p≤.001) of the variance explained for anxiety and 4% (ΔR^2^=.04, p≤.001) for depression. Both variables positively predicted 6% of anxiety and depression (R^2^adj=.06) with perception of threat of the illness being slightly more explanatory (anxiety: β=0.18, p ≤.001; depression: β=0.20, p≤.001) than age (anxiety: β=0.16, p=.02; depression: β=0.15, p≤.001).

Differences in the perception of the disease according to sociodemographic variables were then analysed. Significant differences were found between all diagnostic groups (F=26.09, p≤.001), with moderate to large effect sizes, with diabetics showing greater threat perception (M=24.39, SD=9.54) than those with SS (M=13.97, SD=9.80, d=1.08) and respiratory disease (M=18.69, SD=9.74, d=0.59), and a greater threat perception among respiratory patients with SS (d=0.49), Likewise, no differences were found between preadolescents and adolescents, but differences were observed depending on gender (t449=-2.18, p=.03). Girls showed a greater perception of illness threat (M = 19.52, SD=9.94) than boys (M=17.41, SD=10.44) with a small effect size (d=0.21).

Finally, in order to facilitate the interpretation of the scores, the main centiles were calculated according to diagnosis and sex, ignoring age groups, given the lack of differences between these groups (Table 3).

**Table 3:** Illness threat perception scales (BIP-Q*) based on diagnosis and sex. Valencia, ESP, Spain, 2016-2018

	Perception of threat of disease (BIP-Q*)						
Centile	DM1^†^ (n^‡^=113)		SS^§^ (n^‡^=135)		Respiratory (n^‡^=241)		Centile
	Boys (n^‡^=62)	Girls (n^‡^=51)	Boys (n^‡^=81)	Girls (n^‡^=54)	Boys (n^‡^=124)	Girls (n^‡^=117)	
90	37	33	26,80	25,50	29,50	34	90
80	29,40	28	18,32	21	25	29	80
70	24,20	25,40	18,32	18,32	23	26	70
60	21	24	17	17	20	23	60
50	18,32	18,32	13	14,50	16	20	50
40	18,32	18,32	11	12	13	17	40
30	18,32	18,32	8,60	9	10	14	30
20	18,32	18,32	7	6	7	12	20
10	13,60	13,20	4,20	3,50	5,50	8	10

* BIP-Q = Disease threat perception questionnaire;

† DM1= Diabetes Mellitus Type 1;

‡ n = Group size;

§ SS = Short stature

## Discussion

The presence of CD in adolescence, whether endocrinological or pneumological, may be perceived as a threat to health and survival^(^ 11 ^)^, which may influence the course and resolution of the disease^(^ 6 ^,^ 8 ^)^. It is therefore necessary to have instruments for its measurement^(^ 12 ^-^ 13 ^)^, and the BIP-Q is the most widely used^(^ 2 ^,^ 17 ^,^ 20 ^)^,

However, to date, psychometric properties have not been analysed in adolescents with CD, or in the specific case of Spain. Our study therefore aims to analyze the psychometric properties of BIP-Q in a sample of 510 adolescents with CD.

After analysing the psychometric properties, the final number of items was reduced to 5, thereby obtaining the reduced version presented in this study that presents adequate psychometric properties. Although the scale itself was already reduced, it was necessary to eliminate items because when analyzing the psychometric properties in our study sample, the adjustment indices were not adequate, and as such the reliability and validity of the questionnaire improved significantly after items 3, 4 and 7 were eliminated. As regards the dimensionality of the scale^(^ 1 ^,^ 17 ^,^ 20 ^)^, although both the unifactorial and the bifactorial models seem adequate, and the unifactorial model generally seems better. The unifactorial solution has been chosen because after analysing the overall results obtained in both the reliability analysis and the validity analysis (exploitative and confirmatory factor), the reliability improves by eliminating the items loaded below .40 ranging from .56 with 8 items, and .76 with 5 items, which is considered acceptable in the unifactorial model. However, scores below .70 are obtained for the bifactorial solution in both the original version and the 5-item version proposed in this study.

As for criteria validity, as suggested by the literature^(^ 1 ^-^ 3 ^)^, the main predictor of anxiety- depressive symptomatology is the level of perception of illness threat, followed by age.

Regarding the analysis of the influence of sociodemographic variables with respect to the perception of illness threat, as suggested by previous research^(^ 10 ^)^, there are differences depending on the type of disease, as adolescents with DM1 show higher levels of threat than adolescents with respiratory problems. Likewise, there are no differences according to age, as indicated in the literature^(^ 36 ^)^, but there are differences according to sex, as in the specialized literature^(^ 24 ^)^. Finally, in order to facilitate diagnosis, interpretation scales are presented that can help health professionals to assess the threat perception of their CD patients quickly and easily.

Despite the contributions of this research, it is not without limitations, particularly the sampling procedures for the sample studied are neither probabilistic nor representative of all CD. It contains a higher proportion of patients with respiratory disease, making it difficult to generalize the results found. Finally, although the use of self-reports is a common tool, it can introduce biases due to social desirability.

Future research should use probability sampling, extend the sample to other settings and even include other paediatric diseases while combining other objective or physiological measures.

Nevertheless, the study is of particular interest in view of the lack of studies in the paediatric context and the sample obtained. The use of this instrument in paediatrics is new, since this questionnaire in the Spanish context has only been used in adult populations^(^ 15 ^)^ but not in adolescent populations. The fact that the questionnaire is not specific to a chronic disease as such, but that it allows substitution of terms such as asthma or diabetes, medical treatment by inhalers or insulin when it is completed facilitates its use. It is therefore useful for any pediatric CD, as well as being easy to administer and correct. The BIP-Q questionnaire is therefore a useful and practical tool with which to evaluate the adjustment to disease.

## Conclusion

The results of this study show that the reduced Spanish version of the Disease Perception Questionnaire (BIP-Q) is a reliable, valid instrument with solid psychometric properties, which works effectively in the evaluation of the threat perception of pediatric chronic diseases
